# 5*H*-1-Benzothio­pyrano[2,3-*b*]pyridin-5-one

**DOI:** 10.1107/S1600536808024628

**Published:** 2008-08-06

**Authors:** Muhammad Naeem Khan, M. Nawaz Tahir, Misbahul Ain Khan, Islam Ullah Khan, Muhammad Nadeem Arshad

**Affiliations:** a Applied Chemistry Research Center, PCSIR Laboratories Complex, Lahore 54600, Pakistan, and PhD Scholar, Department of Chemistry, Islamia University, Bahawalpur, Pakistan; b University of Sargodha, Department of Physics, Sargodha, Pakistan; c Department of Chemistry, Islamia University, Bahawalpur, Pakistan; d Government College University, Department of Chemistry, Lahore, Pakistan

## Abstract

Mol­ecules of the title compound, C_12_H_7_NOS, with one half-mol­ecule in the asymmetric unit, are disordered about a crystallographic centre of inversion. Refinement showed that the C=O group is disordered with the S atom and the N atom is disordered over four positions. Adjacent mol­ecules are connected through C—H⋯O hydrogen bonds and π⋯π inter­actions (centroid–centroid distances of 3.635 and 3.858 Å).

## Related literature

For related literature, see: Hidetoshi (1997 ▶); Khan *et al.* (2008 ▶); Mann & Reid (1952 ▶).
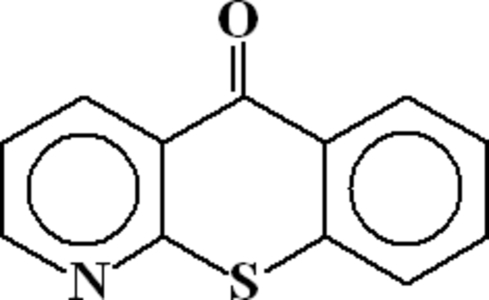


## Experimental

### 

#### Crystal data

C_12_H_7_NOS
*M* *_r_* = 213.26
Monoclinic, 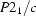

*a* = 7.7308 (18) Å
*b* = 3.8585 (9) Å
*c* = 15.771 (3) Å
β = 99.333 (9)°
*V* = 464.20 (18) Å^3^
*Z* = 2
Mo *K*α radiation
μ = 0.31 mm^−1^
*T* = 296 (2) K
0.25 × 0.06 × 0.04 mm

#### Data collection

Bruker Kappa APEXII CCD diffractometer
Absorption correction: multi-scan (*SADABS*; Bruker, 2005 ▶) *T*_min_ = 0.977, *T*_max_ = 0.987
5384 measured reflections
1189 independent reflections
822 reflections with *I* > 2σ(*I*)
*R*_int_ = 0.034

#### Refinement

*R*[*F* ^2^ > 2σ(*F* ^2^)] = 0.070
*wR*(*F* ^2^) = 0.179
*S* = 1.17
1189 reflections
83 parameters
H-atom parameters constrained
Δρ_max_ = 0.62 e Å^−3^
Δρ_min_ = −0.26 e Å^−3^

### 

Data collection: *APEX2* (Bruker, 2007 ▶); cell refinement: *APEX2*; data reduction: *SAINT* (Bruker, 2007 ▶); program(s) used to solve structure: *SHELXS97* (Sheldrick, 2008 ▶); program(s) used to refine structure: *SHELXL97* (Sheldrick, 2008 ▶); molecular graphics: *ORTEP-3 for Windows* (Farrugia, 1997 ▶) and *PLATON* (Spek, 2003 ▶); software used to prepare material for publication: *WinGX* (Farrugia, 1999 ▶) and *PLATON*.

## Supplementary Material

Crystal structure: contains datablocks global, I. DOI: 10.1107/S1600536808024628/bt2756sup1.cif

Structure factors: contains datablocks I. DOI: 10.1107/S1600536808024628/bt2756Isup2.hkl

Additional supplementary materials:  crystallographic information; 3D view; checkCIF report

## Figures and Tables

**Table 1:** Hydrogen-bond geometry (Å, °)

*D*—H⋯*A*	*D*—H	H⋯*A*	*D*⋯*A*	*D*—H⋯*A*
C5—H5⋯O1^i^	0.93	2.53	3.286 (7)	139

Symmetry code: (i) 
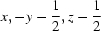
.

**Fig. 1.**
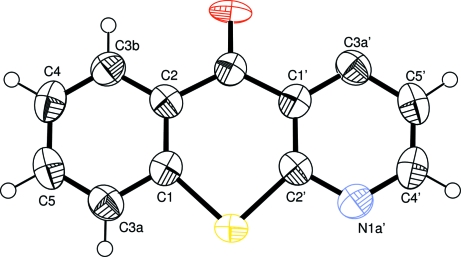
ORTEP-3 for Windows (Farrugia, 1997) drawing of the title compound. The symmetry related atoms are shown by putting ' on the names. The displacement ellipsoids are drawn at the 50% probability level. H-atoms are shown by small circles of arbitrary radii.

## supplementary crystallographic information

### Comment

In continuation of our studies of pyridine containing heterocyclic compounds (Khan, *et al.*, 2008), the title compound has been synthesized. As the molecule is located on a centre of inversion the thio (S1) and carbonyl group (C6=O1) are disordered over two sites with 50% occupancy. For the N atom four different positions were found with an occupancy factor of 0.25. Adjacent molecules are linked to each other through intermolecular H-bonding of C—H···O type (Table 1). In addition, there are π···π-interactions between the the adjacent molecules. The centroid of the ring composed by C1, C2, C3A, C4, C5, and N1B is at 3.635Å from the centroid of the central ring and at 3.858Å from the centroid of its symmetry equivalent (symmetry operator for both centroids: *x*, *y*-1, *z*)

### Experimental

A mixture of 2-chloronicotinic acid (1.57 g, 10 mmol) and thiophenol (2 ml) was heated under reflux for two hours to produce 2-(phenylsulfanyl) pyridine-3-carboxylic acid (Mann & Reid,   1952). The pollyphosforic acid (PPA) (Hidetoshi, 1997) was used to obtain 5*H*-benzothiopyrano[2,3-*b*]pyridin-5-one   after cyclization. Crystals suitable for X-ray diffraction were obtained by cooling the saturated solution of the title compound in chloroform.

### Refinement

For the molecule  is disordered, during refinement EXYZ and EADP were used for N1A, C3B and N1B, C3A. The occupancy factors   for N1A and N1B refined to 0.231 (4) and 0.269 (4), respectively. Thus, they were fixed to 0.25 whereas for C3A and C3B the site occupation factors were fixed to  0.75.

The H atoms were positioned geometrically, with C—H = 0.93 Å, *U*_iso_(H) = 1.2*U*_eq_(C) and constrained to ride on their parent atoms.

### Crystal data

**Table:**

C_12_H_7_NOS	*F*_000_ = 220
*M**_r_* = 213.26	*D*_x_ = 1.525 Mg m^−^^3^
Monoclinic, *P*2_1_/*c*	Mo *K*α radiation λ = 0.71073 Å
Hall symbol: -P 2ybc	Cell parameters from 1189 reflections
*a* = 7.7308 (18) Å	θ = 2.6–28.7º
*b* = 3.8585 (9) Å	µ = 0.31 mm^−^^1^
*c* = 15.771 (3) Å	*T* = 296 (2) K
β = 99.333 (9)º	Needle, light yellow
*V* = 464.20 (18) Å^3^	0.25 × 0.06 × 0.04 mm
*Z* = 2	

### Data collection

**Table:**

Bruker Kappa APEXII CCD diffractometer	1189 independent reflections
Radiation source: fine-focus sealed tube	822 reflections with *I* > 2σ(*I*)
Monochromator: graphite	*R*_int_ = 0.034
Detector resolution: 7.5 pixels mm^-1^	θ_max_ = 28.7º
*T* = 296(2) K	θ_min_ = 2.6º
ω scans	*h* = −10→10
Absorption correction: multi-scan(SADABS; Bruker, 2005)	*k* = −5→3
*T*_min_ = 0.977, *T*_max_ = 0.987	*l* = −21→21
5384 measured reflections	

### Refinement

**Table:**

Refinement on *F*^2^	Secondary atom site location: difference Fourier map
Least-squares matrix: full	Hydrogen site location: inferred from neighbouring sites
*R*[*F*^2^ > 2σ(*F*^2^)] = 0.070	H-atom parameters constrained
*wR*(*F*^2^) = 0.179	*w* = 1/[σ^2^(*F*_o_^2^) + (0.064*P*)^2^ + 0.435*P*] where *P* = (*F*_o_^2^ + 2*F*_c_^2^)/3
*S* = 1.17	(Δ/σ)_max_ < 0.001
1189 reflections	Δρ_max_ = 0.62 e Å^−^^3^
83 parameters	Δρ_min_ = −0.26 e Å^−^^3^
Primary atom site location: structure-invariant direct methods	Extinction correction: none

### Special details

**Table:**

Geometry. All e.s.d.'s (except the e.s.d. in the dihedral angle between two l.s. planes) are estimated using the full covariance matrix. The cell e.s.d.'s are taken into account individually in the estimation of e.s.d.'s in distances, angles and torsion angles; correlations between e.s.d.'s in cell parameters are only used when they are defined by crystal symmetry. An approximate (isotropic) treatment of cell e.s.d.'s is used for estimating e.s.d.'s involving l.s. planes.
Refinement. Refinement of *F*^2^ against ALL reflections. The weighted *R*-factor *wR* and goodness of fit *S* are based on *F*^2^, conventional *R*-factors *R* are based on *F*, with *F* set to zero for negative *F*^2^. The threshold expression of *F*^2^ > σ(*F*^2^) is used only for calculating *R*-factors(gt) *etc*. and is not relevant to the choice of reflections for refinement. *R*-factors based on *F*^2^ are statistically about twice as large as those based on *F*, and *R*- factors based on ALL data will be even larger.

### Fractional atomic coordinates and isotropic or equivalent isotropic displacement parameters (Å^2^)

**Table:**

	*x*	*y*	*z*	*U*_iso_*/*U*_eq_	Occ. (<1)
C6	0.3852 (15)	0.001 (3)	0.0576 (7)	0.044 (2)	0.50
O1	0.2856 (9)	−0.006 (2)	0.1093 (4)	0.083 (3)	0.50
C3B	0.1527 (4)	−0.2668 (8)	−0.05322 (19)	0.0508 (7)	0.75
H3B	0.0739	−0.2649	−0.0145	0.061*	0.75
N1B	0.3839 (4)	−0.2689 (8)	−0.17028 (19)	0.0530 (8)	0.25
S1	0.6613 (4)	−0.0009 (10)	−0.08029 (19)	0.0481 (6)	0.50
C3A	0.3839 (4)	−0.2689 (8)	−0.17028 (19)	0.0530 (8)	0.75
H3A	0.4605	−0.2676	−0.2100	0.064*	0.75
N1A	0.1527 (4)	−0.2668 (8)	−0.05322 (19)	0.0508 (7)	0.25
C1	0.4368 (4)	−0.1412 (8)	−0.08724 (19)	0.0444 (7)	
C2	0.3204 (4)	−0.1406 (8)	−0.02885 (17)	0.0437 (7)	
C4	0.1027 (4)	−0.3937 (9)	−0.1335 (2)	0.0577 (9)	
H4	−0.0102	−0.4803	−0.1495	0.069*	
C5	0.2178 (5)	−0.3955 (9)	−0.1919 (2)	0.0570 (9)	
H5	0.1816	−0.4842	−0.2467	0.068*	

### Atomic displacement parameters (Å^2^)

**Table:**

	*U*^11^	*U*^22^	*U*^33^	*U*^12^	*U*^13^	*U*^23^
C6	0.045 (6)	0.038 (3)	0.047 (6)	0.002 (4)	0.002 (3)	0.008 (4)
O1	0.073 (4)	0.134 (6)	0.053 (4)	−0.023 (4)	0.047 (3)	−0.015 (4)
C3B	0.0496 (16)	0.0468 (16)	0.0563 (17)	−0.0002 (13)	0.0099 (13)	0.0016 (13)
N1B	0.0632 (19)	0.0482 (17)	0.0476 (15)	0.0023 (14)	0.0090 (13)	−0.0010 (13)
S1	0.0438 (17)	0.0620 (11)	0.0411 (16)	−0.0040 (13)	0.0145 (8)	−0.0032 (13)
C3A	0.0632 (19)	0.0482 (17)	0.0476 (15)	0.0023 (14)	0.0090 (13)	−0.0010 (13)
N1A	0.0496 (16)	0.0468 (16)	0.0563 (17)	−0.0002 (13)	0.0099 (13)	0.0016 (13)
C1	0.0434 (14)	0.0371 (14)	0.0512 (16)	0.0028 (12)	0.0031 (12)	0.0058 (12)
C2	0.0522 (16)	0.0361 (14)	0.0420 (14)	0.0059 (12)	0.0049 (12)	0.0037 (11)
C4	0.0501 (18)	0.0507 (19)	0.066 (2)	−0.0058 (14)	−0.0082 (15)	0.0034 (15)
C5	0.069 (2)	0.0495 (18)	0.0474 (17)	0.0000 (16)	−0.0075 (15)	−0.0048 (14)

### Geometric parameters (Å, °)

**Table:**

C6—O1	1.209 (9)	S1—C2^i^	1.791 (4)
C6—C2	1.480 (13)	S1—C1	1.803 (4)
C6—C1^i^	1.481 (13)	C1—C2	1.388 (4)
C3B—C4	1.355 (4)	C1—C6^i^	1.481 (13)
C3B—C2	1.380 (4)	C2—S1^i^	1.791 (4)
C3B—H3B	0.9300	C4—C5	1.380 (5)
N1B—C5	1.364 (5)	C4—H4	0.9300
N1B—C1	1.398 (4)	C5—H5	0.9300
			
O1—C6—C2	117.2 (11)	C6^i^—C1—S1	15.5 (4)
O1—C6—C1^i^	117.1 (11)	C3B—C2—C1	119.7 (3)
C2—C6—C1^i^	125.7 (7)	C3B—C2—C6	123.3 (5)
C4—C3B—C2	120.0 (3)	C1—C2—C6	117.0 (5)
C4—C3B—H3B	120.0	C3B—C2—S1^i^	107.4 (2)
C2—C3B—H3B	120.0	C1—C2—S1^i^	132.9 (3)
C5—N1B—C1	118.7 (3)	C6—C2—S1^i^	15.9 (4)
C2^i^—S1—C1	94.30 (18)	C3B—C4—C5	120.6 (3)
C2—C1—N1B	120.0 (3)	C3B—C4—H4	119.7
C2—C1—C6^i^	117.3 (5)	C5—C4—H4	119.7
N1B—C1—C6^i^	122.7 (5)	N1B—C5—C4	120.9 (3)
C2—C1—S1	132.8 (3)	N1B—C5—H5	119.6
N1B—C1—S1	107.2 (2)	C4—C5—H5	119.6
			
C5—N1B—C1—C2	−0.8 (5)	S1—C1—C2—C6	0.2 (6)
C5—N1B—C1—C6^i^	179.5 (6)	N1B—C1—C2—S1^i^	−179.5 (3)
C5—N1B—C1—S1	179.4 (3)	C6^i^—C1—C2—S1^i^	0.2 (6)
C2^i^—S1—C1—C2	−0.2 (4)	S1—C1—C2—S1^i^	0.2 (6)
C2^i^—S1—C1—N1B	179.6 (2)	O1—C6—C2—C3B	2.2 (15)
C2^i^—S1—C1—C6^i^	0(2)	C1^i^—C6—C2—C3B	−179.7 (7)
C4—C3B—C2—C1	0.6 (5)	O1—C6—C2—C1	−178.2 (10)
C4—C3B—C2—C6	−179.8 (6)	C1^i^—C6—C2—C1	−0.1 (12)
C4—C3B—C2—S1^i^	−179.7 (3)	O1—C6—C2—S1^i^	2.0 (11)
N1B—C1—C2—C3B	0.1 (4)	C1^i^—C6—C2—S1^i^	−180 (3)
C6^i^—C1—C2—C3B	179.7 (6)	C2—C3B—C4—C5	−0.5 (5)
S1—C1—C2—C3B	179.8 (3)	C1—N1B—C5—C4	0.9 (5)
N1B—C1—C2—C6	−179.6 (6)	C3B—C4—C5—N1B	−0.2 (5)
C6^i^—C1—C2—C6	0.1 (11)		

Symmetry codes: (i) −*x*+1, −*y*, −*z*.

### Hydrogen-bond geometry (Å, °)

**Table:**

*D*—H···*A*	*D*—H	H···*A*	*D*···*A*	*D*—H···*A*
C5—H5···O1^ii^	0.93	2.53	3.286 (7)	139

Symmetry codes: (ii) *x*, −*y*−1/2, *z*−1/2.
